# Access to cancer preventive care and program considerations for people experiencing homelessness across four European countries: an exploratory qualitative study

**DOI:** 10.1016/j.eclinm.2023.102095

**Published:** 2023-07-20

**Authors:** Tobias Schiffler, Christina Carmichael, Lee Smith, Ascensión Doñate-Martínez, Tamara Alhambra-Borrás, Miguel Rico Varadé, Jaime Barrio Cortes, Matina Kouvari, Pania Karnaki, Maria Moudatsou, Ioanna Tabaki, Alejandro Gil-Salmeron, Igor Grabovac

**Affiliations:** a Centre for Public Health, Department of Social and Preventive Medicine, Medical University of Vienna, Vienna, Austria; b Centre for Health, Performance and Wellbeing, Anglia Ruskin University, Cambridge, UK; c Polibienestar Research Institute, University of Valencia, Valencia, Spain; d General Directorate of Social Services, Council of Family, Youth and Social Policy, Community of Madrid, Madrid, Spain; e Foundation for Biosanitary Research and Innovation in Primary Care, Madrid, Spain; f Institute of Preventive Medicine, Environmental and Occupational Health Prolepsis, Athens, Greece; g PRAKSIS – Programs of Development, Social Support and Medical Cooperation, Athens, Greece; h International Foundation for Integrated Care, Oxford, UK

**Keywords:** Homeless persons, Cancer, Neoplasms, Prevention, Healthcare disparities, Qualitative study

## Abstract

**Background:**

People experiencing homelessness (PEH) have a higher prevalence of adverse health outcomes and premature mortality compared to the non-homeless population. These include a higher burden of cancer and cancer-specific morbidity and mortality—outcomes that may be a consequence of significant barriers to accessing primary and secondary prevention and community health services. This study aimed to better comprehend the health needs and barriers to accessing preventive cancer care for PEH across four European countries as well as necessary considerations for developing interventions around cancer prevention for this population.

**Methods:**

In this exploratory qualitative study, 69 semi-structured interviews were conducted across Austria, Greece, Spain, and the UK, with a sample comprising 15 professionals working in homelessness support services, 19 health professionals, and 35 PEH. Interviews took place between August 1 and October 31, 2021, and data were analysed inductively and iteratively following a thematic approach.

**Findings:**

Findings were organised into two overarching themes: (1) Experiences and understanding of cancer prevention and treatment and (2) Considerations for program interventions. While cancer was a significant worry among PEH across all settings, they generally had minimal knowledge and understanding of cancer symptoms and prevention. Specific programs for cancer prevention for PEH were described as almost non-existent. Health professionals in some settings indicated that cancer in PEH was often missed in the early stages and instead diagnosed when the severity of symptoms intensified.

**Interpretation:**

Overall, our findings indicate many commonalities in the health needs of PEH and the barriers they face when they seek access to cancer-specific healthcare services in the European context.

**Funding:**

This study received funding from the 10.13039/501100007601European Union’s Horizon 2020 Research and Innovation Programme under GA 965351.

**Research in context**

**Evidence before this study**

People experiencing homelessness (PEH) have a higher prevalence of adverse health outcomes and premature mortality when compared to people not experiencing homelessness, and they experience significant barriers to accessing healthcare, including primary and secondary prevention services and programs. Prior to recruiting participants for this study, we searched PubMed for articles on June 15, 2021, using the search term “(homeless∗) AND (cancer∗) AND (prevent∗) AND (access∗)” which yielded 24 results of which 15 were not relevant to our scope, and nine investigated the issue of interest in other healthcare settings and were mainly quantitative studies; an updated search in PubMed on June 2, 2023, yielded no additional results. As shown in a recently published review, data on cancer health and cancer screening in PEH and related strategies to address barriers they experience when seeking such services are still scarce.

**Added value of this study**

This study is among the first to provide comprehensive insight into the perceived accessibility of existing cancer prevention and screening services on a European level from the point of view of PEH and professionals who work with this population. Moreover, it gives an overview of what a program intervention should take into consideration when aiming at improving preventive cancer care for PEH.

**Implications of all the available evidence**

This qualitative study was conducted within the framework of the CANCERLESS project (“*Cancer prevention and early detection among the homeless population in Europe: Co-adapting and implementing the Health Navigator Model*”) financed by the EU Horizon 2020 program. Overall, available evidence indicates that throughout the European context there exists a consistency in the cancer-specific health needs of PEH as well as in the barriers and facilitators they experience when accessing healthcare services. The findings of this study will substantially inform the components of a novel, evidence-based, and person-centred healthcare model—the Health Navigator Model—that will address the growing health and social disparities in PEH and promote their timely access to cancer prevention and screening services.

## Introduction

People experiencing homelessness (PEH) represent an underserved and marginalised population with a higher prevalence of adverse medical conditions, including infectious diseases, substance-related disorders, and mental ill-health, compared to the general population.^1^ Data from the UK indicates that PEH have an average life expectancy of 47 years, being 30 years lower than that of the general population.^2^^,^^3^

Cancer is one of the most prevalent causes of death in Europe.^4^ The most common types associated with the highest burden of cancer-specific mortality are lung, colorectal, and breast. Indeed, while Europe holds around 9% of the world population, it carries around one-quarter of the global cancer disease burden, despite ongoing screening and prevention campaigns and strategies. At present, cancer is the second most common cause of death among PEH, with cancer-related mortality twice that of the general adult population in countries in the Global North.^5^^,^^6^ In a large-scale cohort study with a sample comprising 60,092 PEH in Massachusetts, cancer accounted for 15.2% of all deaths during a study period of 15 years.^7^ This higher disease burden can be explained by exposure to a variety of risk factors, including a higher prevalence of cancer risk behaviours (e.g., tobacco or alcohol use), infectious diseases, and malnutrition, and the existence of barriers when accessing complex and fragmented health and social care systems.^8^ However, it is essential to acknowledge that these barriers not only impact PEH but also affect other vulnerable populations.

Existing evidence has highlighted that PEH often have little or no access to primary and secondary prevention strategies or community health services, with most healthcare utilisation happening within acute healthcare settings.^9^ As a result, it has been found that many deaths among PEH are from preventable and treatable medical conditions, such as heart disease, pneumonia, or cancer.^6^ While there is potential for such conditions to be addressed through early and effective healthcare services tailored for PEH, the social and financial exclusion they experience often hinders them from accessing these services.^8^^,^^10^ The literature concerning cancer screening access and outcomes among PEH is notably limited, particularly in comprehending their distinct challenges and disparities in this context.^11^^,^^12^ Due to this limited knowledge, it remains unclear which factors contribute to the low number of PEH screened and treated for cancer and what strategies might be most effective to increase the use of cancer preventive services among this population.^13^

This study sought to gain a greater understanding of PEH's concerns, care needs, and preferences regarding cancer prevention, screening, and cancer care from the point of view of both PEH and professionals active in health and social care across four different European healthcare systems. It aimed to explore the barriers and facilitators to primary and secondary cancer preventive healthcare faced by PEH and understand the necessary considerations for designing and implementing targeted cancer prevention care within homeless populations. Moreover, our objective was to better comprehend the existing practices and knowledge surrounding cancer prevention among PEH and social and healthcare professionals while pinpointing key factors that must be taken into account in order to enhance cancer prevention efforts for this vulnerable population.

## Methods

### Design

Following an exploratory qualitative research design, data were collected through semi-structured interviews with PEH, with and without direct experience of cancer, and relevant health and social care professionals. Interviews were conducted in field settings across four European countries—Austria, Greece, Spain, and the UK—by a multidisciplinary team of researchers from a total of seven partner organisations between August 1 and October 31, 2021.

Employing a qualitative design allowed for an in-depth exploration of the topic area while ensuring participants' perspectives, experiences, and language remained central through the analytical process and in developing the key themes. The flexibility offered by a qualitative approach guaranteed that participants were granted a level of freedom and power to share what they felt to be most important or relevant, which is particularly important given that PEH are often highly marginalised. Moreover, given the topic's sensitive nature, the chosen method needed to allow for rapport-building between researchers and participants. In this regard, it has been noted that the non-prescriptive nature of the qualitative interview format means participants are more likely to feel comfortable revealing their experiences and opinions.^14^

### Participants

All participants had to be at least 18 years of age to be eligible for inclusion in our study. Participants were classified as experiencing homelessness according to the European Typology of Homelessness and Housing Exclusion (ETHOS).^15^ PEH, both with and without a cancer diagnosis, as well as cancer survivors experiencing homelessness were included.

Participants from the group of professionals had to be occupied in the field of healthcare (e.g., nurses, oncologists, primary care physicians, psychiatrists) or psycho-social care (e.g., professionals working in organisations for cancer patients, social workers, and support workers).

Participants were recruited in all partner countries with the assistance of relevant organisations, such as accommodation providers, health services, advisory services, or day centres. The study teams disseminated information via verbal and email contact about the purpose and objectives of the study and the inclusion criteria of participation to staff representatives (e.g., service managers), who then helped establish contacts between researchers and participants they deemed appropriate for inclusion. Researchers finally selected participants following the study eligibility criteria. No incentive was offered to participants for taking part in an interview.

### Data collection and analysis

Interviews generally took place within the premises of the service or organisation with which an individual participant was associated (e.g., a day centre for PEH or a health service) and were conducted in private or semi-private settings (e.g., a private office or a quiet area of a shared social space). Where necessary because of COVID-19-related restrictions, some interviews also took place remotely via video call platforms.

Five question schedules were used to guide the central part of the interviews and encourage consistency in the cross-national data collection (see Appendix 1). Question schedules were designed to be open-ended and exploratory and were developed in consultation with professionals from a homelessness organisation to ensure the suitability of language and content. An iterative process involving representatives from all piloting sites was undertaken to ensure that every voice was heard and incorporated into the finalised document. Specific schedules were designed for both participant groups covering the same broad general health and cancer-related topics. Cancer-specific areas included current knowledge and practices relating to cancer prevention, direct experiences of cancer prevention and treatment, suggestions, priorities, and perceived challenges in improving cancer prevention and care pathways. All interviews were audio-recorded and transcribed verbatim in their respective languages, either manually or using appropriate software, before being internally checked for quality. The completed transcripts were analysed according to the inductive thematic approach by Saldaña,^16^ aided by either NVivo v12 (https://lumivero.com/products/nvivo/) or Atlas.ti v8 (https://atlasti.com/). Researchers worked through the data systematically, attaching labels of a few words (codes) to capture meaning in the text. These codes were then reviewed and merged, first into tentative categories and then overarching themes, allowing the abundance of codes identified through the initial analysis to be condensed and synthesised. While the initial coding process was completed country by country, partners from all countries met regularly to discuss the data analysis and the final themes were collectively agreed upon.

### Ethical practice

Before the start of the interviews, participants were supplied with an information leaflet about the study (participant information sheet) and offered the opportunity to ask questions. In all cases, participants were reminded that participation was voluntary and that they did not need to answer any questions that made them feel uncomfortable. Full informed consent was sought from and provided by all participants both verbally and through a signed consent form. Data have been stored securely, and all files have been encrypted and stored on computers requiring password access, avoiding duplicates. All interviews were transcribed, omitting identifiable details, and both interview transcripts and audio files were labelled using reference codes rather than names. The interview consent forms have been stored securely and separately, linked to their respective transcripts via an anonymous reference code. Throughout this paper, quotations have been carefully considered to ensure they do not reveal an inappropriate level of detail about specific participants.

While each partner country obtained approval from its designated ethical review board, the Ethics Committee of the Medical University of Vienna served as the lead ethics committee for the project and approved this study (1702/2021).

### Role of the funding source

The funder of the study had no role in the study design, data collection, data analysis, data interpretation, or writing of this report. All authors had full access to all the data in the study and accept responsibility for the decision to submit for publication.

## Results

Sixty-nine participants took part in interviews across the four partner countries, with a sample breakdown of overall 35 PEH—of whom 26 did not have direct experience of cancer, six were living with cancer (one person each with breast cancer, cervical cancer, lung cancer, skin cancer, uterine cancer, and non-specified cancer), two were cancer survivors (one person each having experienced breast cancer and colon cancer), and one was living with oesophageal cancer while being an anal cancer survivor—as well as 15 psycho-social care professionals (e.g., social workers, (peer) support workers, professionals working in organisations for cancer patients) and 19 health professionals (9 primary care physicians, six nurses, two oncologists, and two psychiatrists). Detailed information about PEH and professionals who participated in the study can be found in Table 1 and Table 2, respectively.

**Table 1:** Participant characteristics—People Experiencing Homelessness.

Characteristic	n (%)
**Age, in years**	
18–39	5 (14)
40–59	24 (69)
≥60	6 (17)
**Sex**	
Female	13 (37)
Male	22 (63)
**Ethnicity**	
Spanish	7 (20)
British	6 (17)
Greek	5 (14)
Austrian	2 (6)
Congolese (DRC)	2 (6)
Arabic	1 (3)
Bangladeshi	1 (3)
Bolivian	1 (3)
Finnish	1 (3)
Georgian	1 (3)
Iranian	1 (3)
Iraqi	1 (3)
Moroccan	1 (3)
Pakistani	1 (3)
Senegalese	1 (3)
Serbian	1 (3)
Slovakian	1 (3)
Uruguayan	1 (3)
**Housing circumstances** (**ETHOS category)**^15^	
1.1. Public space or external space	3 (9)
2.1. Night shelter	1 (3)
3.1. Homeless hostel	14 (40)
3.2. Temporary accommodation	2 (6)
7.2. Supported accommodation for formerly homeless people	4 (11)
8.1. Temporarily with family/friends	5 (14)
8.2. No legal (sub)tenancy	1 (3)
11.2. Non-conventional building	2 (6)
11.3. Temporary structure	2 (6)
13.1. Highest national norm of overcrowding	1 (3)
**Cancer-specific health condition**	
PEH living with cancer	7 (19)
Cancer survivors	3 (8)
No diagnosed cancer	26 (72)
**Location of interview**	
Madrid, Spain	11 (31)
Vienna, Austria	8 (23)
Athens, Greece	7 (20)
Norfolk, UK	6 (17)
Piraeus, Greece	3 (9)

ETHOS: European Typology of Homelessness and Housing Exclusion; PEH: People experiencing homelessness.

**Table 2:** Participant characteristics—Numbers of Health and Social Care Professionals.

	Austria	Greece	Spain	UK	*Total*
Social/support worker	5	1	2	5	13
Nurse		1	2	3	6
General Practitioner	5				5
Primary Care Physician		1	2	1	4
Oncologist	1	1			2
Child and Adolescent Psychiatrist		1			1
Patients' Organization Professional		1			1
Psychiatrist		1			1
Sociologist		1			1
***Total***	11	8	6	9	34

The overarching themes derived from the data were (1) “Experiences and understanding of cancer prevention and treatment”, subdivided into the categories “Limited knowledge and awareness of cancer prevention among PEH” and “Non-priority of cancer prevention”, and (2) “Considerations for designing and delivering cancer prevention programs”, subdivided into the categories “Building trusting relationships and tailoring services to meet the needs of PEH” and “Improving accessibility of prevention”. The findings of our thematic analysis are presented below.

### Experiences and understanding of cancer prevention and treatment

#### Limited knowledge and awareness of cancer prevention among PEH

Considering their respective situations, participants did not receive information from healthcare professionals on cancer prevention. At the same time, it was also clear that most participants experiencing homelessness were unaware or had not been involved in any national cancer prevention program (e.g., screening). Participants also reported a lack of resources for preventive efforts in PEH, highlighting problems in national or mainstream screening and health education programs, which are not designed to reach this population:

“*No, I have never been informed about cancer prevention. I don’t know how to prevent it. If I get checked up, then I might be able to see if I have it or not.*”

(PEH, Greece)

“*As far as I know, no. I mean, for the patients I work with [PEH] I do not know any cancer prevention programs. If a person who lives on the street is identified and has cancer, it is because we have insisted and insisted … no one has contacted us about any cancer treatment program.*”

(Primary care physician, Spain)

In this regard, participants pointed out that without a clear understanding of cancer care pathways, the process can be exceptionally daunting for PEH, as there exists a severe lack of coordination and continuity in care:

“*I had to go for a screening myself because I had a lump in the middle of my chest, I’ve still got it now, but they don’t know what it is [Interviewer: So, do you know what happens next?] No, I’m just basically, you know, left in the lurch at the moment.*”

(PEH, UK)

Although participants across groups recognised the seriousness of cancer among PEH, many participants from the social care sector and those who experienced homelessness felt that their awareness and knowledge of cancer prevention and symptoms were limited. However, individuals with a direct personal experience of living with cancer expressed a higher level of cancer awareness, and thus, the lack of knowledge primarily applied to those without any previous firsthand cancer experience. Where participants did share their understanding, this was generally relatively vague and based on personal experiences or internet searches:

“*Look, overall, they [health professionals] say prevention saves lives. Apart from that, I have no general knowledge of the topic because I never bothered, or it did not concern me until now. It is good to be aware of these things. It is even harder if you don’t have access to any of this. I just think information is the most important thing from start to finish.*”

(PEH, Greece)

“*I don’t think I would have a clue how to spot the symptoms. There’s no wrong or right way that we’ve been taught, and we need to be aware, but none of us have got that knowledge of it.*”

(PEH, UK)

While some participants felt that combining regular healthcare with cancer screening was something to consider, healthcare professionals stressed that the traditional health checks were unsuitable for cancer prevention and that there were no effective screening programs in place for PEH. However, some respondents were aware of PEH living with cancer being provided treatment (e.g., radiotherapy) partly by local homelessness care facilities, and a few of those who had directly experienced cancer highlighted that the costs of treatment were a significant issue for them. PEH experiencing cancer also described that they received very limited support at the point of cancer diagnosis.

#### Non-priority of cancer prevention

Both professionals and PEH themselves explained that cancer screenings do not constitute a priority for PEH compared to meeting other, more immediate needs, such as accommodation, food, work, and clothing. It was also emphasised that PEH might not prioritise preventive examinations or even attend appointments following cancer diagnosis due to their poor mental health, mainly when living on the street. Participants across groups noted that cancer prevention is irrelevant if acute problems are not dealt with first. However, they recognised that financial issues had to be clarified at the stage of diagnosis, as cancer was likely to spread by the time care was assured. Incomplete diagnostic procedures and follow-ups were often due to a lack of financial means, unemployment, as well as structural issues such as lack of appropriate housing:

“*So, it is really a luxury to take care of preventive examinations and check-ups when you're still carrying acute problems around with you.*”

(PEH, Austria)

“*Then sometimes, examinations take place whereby the findings are inconclusive because the preparation does not work … but do a laxative preparation for a colonoscopy while living on the street … so, there are things like that. You can offer so many appointments and so many laxatives … if you do not think about it, where do people do that? Then, the medical examination will not work.*”

(General practitioner, Austria)

From the perspective of health professionals, the lack of preventive care meant that cancer diagnosis often occurred by chance at advanced stages of the disease and was only detected because of a specific intervention performed by specialist healthcare organisations and specialists who work with PEH:

“*So, by the time a homeless woman gets an indication that she has breast cancer, it must have already spread because, as you know, it does not cause pain. This is why prevention is essential when it comes to cancer.*”

(Specialist care physician, Greece)

Health and social care professionals emphasised the difficulties associated with developing consistent communication channels with PEH and maintaining continuous engagement with mainstream healthcare services. These difficulties around continuity in care for PEH are related to healthcare systems and individual lifestyles and make ongoing dialogue or treatment challenging, which is why professional participants felt there to be a need for a specific and tailored pathway for cancer prevention:

“*The problem is that I do not know if it can be carried out within the current healthcare system, but what is clear is that we need to create a special pathway for this group of people, not to discriminate against them, but to facilitate … Because these patients [PEH] can have thousands of things at the healthcare centres and hospitals, and if we want them to participate in preventive activities—in this case, related to cancer—then we need them to be within the health system.*”

(Primary care physician, Spain)

### Considerations for designing and delivering cancer prevention programs

#### Building trusting relationships and tailoring services to meet the needs of PEH

Respondents across health and social care sectors felt that in order to work towards low-threshold care for PEH, specialists and staff must be willing to work with this specific population regardless of their physical or mental state. Some of these participants also indicated that broader cooperation with existing services was essential and that more psychological support provision in the network was required. Participants suggested that PEH should be able to access affordable housing and healthcare and that there should be greater clarity from social insurance on therapy coverage. Many participants also recommended a more direct, consistent, and multilingual outreach to ensure greater awareness of available offers to PEH:

“*Well, I would say that communication is the most important challenge here … It is the fact of being on the street, trying to approach these people on the street, trying to know or determine what is happening to them, their pathology … this is complicated, and it is very complex because on the street there are many people who do not want to communicate, they just want you to feed them and leave them alone and go away … then the key and important concept is the communication with the homeless people.*”

(PEH, Spain)

Regarding the delivery of cancer treatment, participants emphasised the need for additional psychological support for PEH from health and social care professionals. In addition, it seems very important to ensure patients experiencing homelessness have access to safe housing and facilities where they can manage their treatment (e.g., access to a fridge to store medicine and running water).

Participants suggested involving a multidisciplinary team comprising health professionals, psycho-social care professionals, and peers (i.e., people with lived experience). Professionals already engaged with and knowledgeable about PEH (e.g., community nurses or social workers) would be best equipped to deliver the program, or that specialist training on the issues surrounding homelessness would be required:

“*Having the language, having knowledge not particularly of services, but of what makes people tick, how they got to where they are, having some mental health understanding, and understanding their own limitations. So, when people have got post-traumatic stress, knowing there is a special clinician to unpack that, it’s not me. I think a knowledge of the lifestyle, so a knowledge of the soup kitchen, knowledge of drugs, paraphernalia, knowledge of how you use drugs, knowledge of drinking habits, knowledge of alcohol abuse, respiratory knowledge, gastro-intestinal knowledge.*”

(Nurse, UK)

Respondents also recognised the need to raise awareness for cancer prevention among healthcare professionals and PEH. Some participants stressed that for healthcare staff to understand the lives and contextual situations faced by a broad range of PEH, they would need to be better informed of the facts on homelessness, and mentioned that peers are a resource that could provide motivation and contextual support:

“*It [prevention measure] should be cheap; it should be informative. It should be cheap if you put it in brochures or information leaflets. By the way, I also do prevention. I inform other homeless people what to look out for, especially when they are homeless in winter. What the dangers are and what problems they can get into as far as health is concerned.*”

(PEH, Austria)

“*Information is the key, the most important thing … I think that is fundamental and more in cancer disease, we are not talking about flu or other small things.*”

(PEH, Spain)

Participants suggested that care providers should not be too intrusive when approaching PEH but instead give them more time and information about the issues they were dealing with. Other strategies proposed by respondents involved having PEH engage in meaningful activities and providing incentives that benefited them, such as nutritious food packages or public transport tickets to motivate participation and inclusion in informational events and continuing care services.

#### Improving accessibility of prevention

Several participants felt strongly that access to preventive examinations (i.e., secondary prevention or primary healthcare) needs to be guaranteed. To facilitate this, it was proposed that medical check-ups could be conducted either in the clinics of homelessness organisations or in partnership with public hospitals/health centres. However, to achieve this sort of direct linkage to hospitals, it was recognised that strong and structured collaboration would be needed between health and social care services.

“*The number one thing to keep in mind is to inform this population what cancer is. The point is to get them into the process of thinking that ‘when I see something strange in my body, I must go to the doctor’. So, in developing a model, first, you inform them, and then you find a way to connect them with health services.*”

(Social worker, Greece)

To meet PEH's needs, it was also suggested that any intervention would need to be proactive in its approach and ‘reach out’ to and encourage engagement from PEH. Several participants also felt that the intervention would need to be longitudinal to allow trusting relationships to develop and remedy the fragmented nature of mainstream healthcare systems. The suitability of the setting was also described as an essential factor for consideration. Along the same lines as the above, participants emphasised that a program delivered in a familiar and trusted space would be most appropriate.

In delivering the intervention, several participants pointed out that a program should begin from the point of improving awareness and providing education to PEH and those that work directly with them. On this point, the importance of using accessible language to deliver critical information was again emphasised:

“*Educate them and show them … Then you’re giving them information to give them control over their own bodies, and to, we all know if you give somebody just that little bit of power back, self-empowerment and it starts to grow.*”

(Social worker, UK)

Finally, participants also felt that it was essential to offer integrated care by supporting patients experiencing homelessness not only at the point of prevention but also during the ongoing treatment and by considering each person's individual support needs and cultural background and appropriately working with them. This integrated care approach would be crucial in regions with a higher prevalence of migrants experiencing homelessness because this population may face additional barriers and challenges in accessing adequate healthcare and support services. Moreover, it was voiced that work would need to be done to ensure more flexible healthcare systems, whereby PEH can avoid unnecessary bureaucratic barriers (e.g., lack of legal documentation) and where there is flexibility in terms of appointment times and locations.

## Discussion

In this qualitative study, we explored both the current practices and future possibilities for cancer prevention and early intervention strategies with PEH, drawing directly on the perspectives from those ‘on the ground’—that is, people with lived experience of homelessness with and without cancer, and professionals working in health and social care services. Overall, what was most notable from the analysis of interview data was that—across four European settings—there appeared to be very little happening by way of cancer prevention and screening activity within the homeless population at present. Although there was limited evidence of tailored or targeted cancer prevention strategies, it was also clear that national campaigns and approaches do not consistently reach the homeless population.^17^ This is despite, as set out above, the disproportionate risk of cancer incidence and cancer-related mortality PEH face.^8^ The data presented in this study demonstrate a high degree of coherence regarding the main themes, as shown above. However, the most prominent differentiating factors primarily revolved around health and social insurance concerns and the prevalence of migrant homelessness within specific geographical contexts. Additionally, disparities were identified in terms of country-specific variations in the characteristics and availability of cancer screening programs, which underscore the importance of contextual factors in shaping healthcare.

There is a dearth of comprehensive data on cancer screening rates among the homeless population across Europe. However, several studies from North America have indicated that uptake is considerably lower than that of the general population.^5^^,^^18^ This aligns with the present study's findings, which found that very few participants recalled any experience with or knowledge of the screening programs available in their respective countries. As reported elsewhere, the findings of this study also indicated a general lack of cancer-based literacy among PEH and, in some cases, frontline (non-clinical) professionals, such as support and social workers.^19^ It was of particular concern that—from the perspective of the health professionals included in the sample—this lack of cancer prevention and screening meant that when a diagnosis was made, it was often at a more advanced stage or when PEH were already in a hospital setting. As cancer is not being spotted among PEH at the early stages but usually only by accident or when more advanced, this contributes to a general distrust of the healthcare system and, thus, low utilisation of these services.^5^ At the same time, the resistance to combining regular healthcare with cancer screening for PEH, as some professional participants expressed it, may stem from resource limitations, the lack of targeted screening programs, the complexity of care coordination, prioritising immediate healthcare needs, and the presence of stigma and discrimination.

In interpreting this data, it is vital to situate the current lack of engagement with cancer prevention on the part of PEH alongside the broader challenges this population often faces in navigating primary healthcare systems.^19^ Even in countries with universal healthcare coverage, populations excluded from the healthcare system, as PEH represent one, are inevitably excluded from cancer preventive care. Without consistent access to primary care, PEH are much less likely to receive information about and invitations to cancer screening and other forms of cancer prevention (e.g., smoking cessation programs).^20^^,^^21^ For example, in the UK, individuals are mainly invited to attend cancer screenings by their primary care physicians, which presents challenges for PEH, who do not regularly engage with primary care, potentially leading to their exclusion from these programs. Moreover, in Spain, the lack of holding an active insurance card is an issue, although the public healthcare system does cover the costs of cancer screening (for breast, colon, and cervical cancer) for vulnerable populations such as PEH. However, most PEH never get the mailed invitation to attend planned screening programs due to lack of a permanent place of residence. In Austria, cancer screening services are only accessible with active health insurance, and in consequence, PEH, who often lack insurance coverage, are excluded from cancer screening programs. Regarding Greece, PEH who have a national insurance number—even when uninsured—can still access preventive screenings through public hospitals, while efforts are made to link those without a national insurance number to medical services through non-governmental organisations. Furthermore, the current cancer screening programs in Austria, Greece, Spain, and the UK lack targeted approaches beyond age and biological sex, despite the higher prevalence of cancer among PEH, which is why a more tailored screening approach is required to address the specific needs of this vulnerable population. Nevertheless, a recent study by Mayo et al.^22^ found that having a primary care provider was the most significant factor associated with cancer screening utilisation.

The findings here also highlight that while the prospect of cancer is a source of worry for PEH, meeting more immediate concerns (such as access to accommodation or emergency healthcare) often takes precedence over engagement with preventive healthcare, mainly if the latter is seen to be challenging to navigate.^19^ There is a substantial lack of policy focusing on cancer preventive mechanisms and cancer-specific healthcare that could be adopted to PEH's needs.^5^^,^^23^ Thus, it is imperative to concurrently support social welfare systems and healthcare systems through policy decisions.

Across our sample, there was consensus regarding the need for tailored and targeted approaches to cancer prevention that account for and are sensitive to PEH's specific needs and circumstances. Central to this were calls to focus interventions on improving health and cancer literacy through accessible education, so PEH feel empowered to manage their health. However, it was also noted that alongside this, there is a need to place an onus on health providers to improve education around marginalised groups and “*embed health literacy communication practices into healthcare provider training programs*”, as noted by Lawrie et al.^19^

In considering how best to deliver cancer prevention to PEH, participants also consistently highlighted the need to build on and utilise the trusting relationships that many PEH already have with specialist support services (such as accommodation facilities and day centres) through the use of proactive ‘in-reach’ and the use of partnership working between health and social care providers from both public and the civil society sectors.^21^ On this point, a growing body of evidence has emphasised the value of ‘navigators’ to facilitate access to cancer screening and provide tailored health education among marginalised populations.^5^ In this sense, navigators have the potential to deliver care coordination, social welfare assistance, and advocacy, support, and outreach work for PEH—intervention categories that have been shown to benefit this population in terms of preventing non-communicable diseases.^24^ While there has only been limited application of such navigation models to homeless communities, existing evidence suggests this tailored community-based intervention could be a promising approach to reducing cancer-based disparities.^25^

This study was conducted within the framework of a cross-national project funded by the Horizon 2020 program of the European Union entitled “Cancer prevention and early detection among the homeless population in Europe: Co-adapting and implementing the Health Navigator Model” (CANCERLESS). CANCERLESS aims to combine the principles of Patient Empowerment and the core features of Patient Navigator models to create a new framework known as the ‘Health Navigator Model’.^25^^,^^26^ This model will be piloted and evaluated with PEH across four European settings to improve cancer prevention access and awareness and reduce health inequalities experienced by this population. As this study was conducted at the co-designing stage of the CANCERLESS project, the findings presented here have been fed into the design of the Health Navigator Model.

Using an exploratory and flexible qualitative design was the main strength of this study as this allowed participants—including a population seldom heard in designing healthcare services–to describe their needs, experiences, and priorities in their terms. While this study included a substantive sample of participants, including those with direct lived experience of homelessness and cancer, the findings should be considered alongside some limitations. In relying on homelessness organisations to access and recruit participants, the experiences and perspectives of ‘hidden’ homeless populations who are not engaged with these services (e.g., people sleeping rough in less visible settings, people who are ‘sofa surfing’, people residing in privately-run hostels) are not represented in this study. Due to challenges with accessing appropriate participants, this study only included a limited sample of health professionals specialised in oncology (n = 3). Additional research involving a more significant number of oncology specialists is needed to understand better current practices for cancer prevention and early intervention among PEH from a clinical standpoint.

It is also recognised that as this data was collected during the COVID-19 pandemic, this could hold implications for both the participant sample—particularly in countries where restrictions were ongoing—and the specific findings of this study. For example, it is possible that participants’ perceptions of the importance of cancer prevention were lessened during this period, with their attention instead focused on the pressing and immediate nature of the pandemic.

Finally, it is essential to recognise that the diversity in national healthcare systems and homelessness service provision may mean that the findings presented here do not indicate what is happening in other contexts. Nonetheless, the commonality and consistency in the experiences and recommendations of participants across the four countries suggest that this data has a high degree of transferability beyond the specific settings where it was collected.

Cancer prevention and screening in PEH remains a neglected area—from the point of view of both healthcare providers and PEH—within healthcare systems, social welfare systems, policies, and academic research, with the existing data in this field currently limited. The qualitative findings from Austria, Greece, Spain, and the UK indicate minimal understanding and experience of cancer prevention among PEH and that developing meaningful and tailored cancer prevention strategies is crucial to address the stark health disparities this population faces across these European countries and beyond. Future research should continue to explore strategies and methods that may increase awareness of and engagement with cancer prevention among PEH, carefully considering the concerns and priorities of this population and those that directly work with them. However, while creating specific models and support for PEH is crucial, it remains essential to prioritise social welfare policies and programs that aim to reduce and ultimately end homelessness.

## Contributors

TS, CC, LS, AGS, and IG contributed to the conceptualisation of this research; TS, CC, ADM, TAB, MRV, JBC, MK, and PK contributed to the data curation; TS, CC, ADM, TAB, MK, and PK were involved in the formal analysis; TS, CC, MM, and IT contributed to the investigation; TS and CC were responsible for methodology, visualisation, and writing of the original draft; CC, ADM, TAB, MK, and PK were supporting with the software; AGS and IG were responsible for funding acquisition; LS, AGS, and IG provided resources, supervision, and validation for this work; and all authors participated in reviewing and editing of the draft, with full access and verification of all study data and assume full responsibility for the decision to submit this work for publication.

## Data sharing statement

The datasets analysed during the current study are not publicly available due to data sensitivity and are available from the corresponding author upon reasonable request.

## Declaration of interests

This publication reflects the author's views. The European Commission is not responsible for any use that may be made of the information it contains.
