# Supplementary material for: Access to cancer preventive care and program considerations for people experiencing homelessness across four European countries: an exploratory qualitative study
Source: eClinicalMedicine. 2023 Jul 20;62:102095. doi: 10.1016/j.eclinm.2023.102095 (PMC10393536; doi:10.1016/j.eclinm.2023.102095)
Supplement: Appendix 1 [file mmc1.pdf]

# Appendix 1. Question Schedules for Interviews with Specific Study Groups.

**Question schedule – People experiencing homelessness.**

1. Demographic questions - age, gender identity, sexual orientation, ethnicity, housing circumstances, long-term health conditions/co-morbidities.

2. How would you describe your health? What impacts, if any, do you feel that your experience of homelessness had on your health?

3. How would you describe your current knowledge/understanding of cancer?
    - *Would you know how to see its symptoms?*
    - *Would you know how to prevent it?*

4. Who would you tend to speak to, or where would you seek help if you were to have a health-related issue or concern? Why do you choose to go there/to them?

5. Can you think of any particularly positive experiences of healthcare since you started to have housing difficulties? If so, what was it about the service/professional that made it a positive experience?

6. In what ways does being homeless make accessing healthcare services more difficult? Can you think of any specific barriers you have faced when seeking support for health-related issues?
    - *Have you ever chosen <u>not</u> to access care or support (or delayed getting access to care or support) for a health-related issue, and if so why was that the case?*

7. Have you ever been invited to attend a screening or appointment relating to cancer prevention, and if so, could you talk me through your experience of this?

8. What do you think would improve health services, and make them more accessible to you and other people experiencing homelessness?

9. What sorts of things do you currently do, or would you like to do to improve your health and wellbeing?
    - *Prompt: Exercise, improve diet, reduce substance use, mindfulness, meaningful activity.*

10. As we work to develop a more tailored model of cancer prevention for people experiencing homelessness, do you think there are any factors that we need to consider?
    - *What could be done to ensure that people experiencing homelessness are appropriately screened for cancer?*

11. Do have anything else you would like to add to this discussion, or any questions you would like to you ask me?

**Question schedule – Cancer patients experiencing homelessness.**

1. Demographic questions - age, gender identity, sexual orientation, ethnicity, housing circumstances, long-term health conditions/issues/co-morbidities.

2. Could you begin by talking me through how you came to be diagnosed with cancer, and the care that you have received from that point on?
    - *What services were you engaged with?*
    - *How was the cancer identified?*
    - *What sort of support/care have you received?*
    - *What sort of support/care are you receiving now?*

3. In what ways have your difficulties with housing impacted your experience of going through cancer treatment?
    - *How easy/hard has it been to get the care that you need?*
    - *Can you think of any specific barriers you faced when seeking care and support for cancer?*

4. Can you think of any particularly helpful or positive experiences you have had with healthcare services? If so, what was it about the service/professional that made it a positive experience?

5. What do you think would improve health, and specifically cancer prevention, services? How could these have been made more accessible to you?
    - *What could be done to ensure that people experiencing homelessness are appropriately screened for cancer?*

6. What sorts of things do you currently do, or would you like to do to improve your health and wellbeing?
    - *Prompt: Exercise, improve diet, reduce substance use, mindfulness, meaningful activity.*

7. As we work to develop a more tailored model of cancer prevention for people experiencing homelessness, do you think there are any particular factors that we need to consider?

8. Do you have anything else you would like to add to this discussion, or any questions you would like to ask me?

**Question schedule – People experiencing homelessness and living beyond cancer (cancer survivors).**

1. Demographic questions - age, gender identity, sexual orientation, ethnicity, housing circumstances, long-term health conditions/issues/co-morbidities.

2. Could you begin by talking me through how you came to be diagnosed with cancer, and the care that you have received from that point on?
    - *What services were you engaged with?*

- *How was the cancer identified?*
- *What sort of support/care have you received?*
- *What sort of support/care are you receiving now?*

3. In what ways have your difficulties with housing impacted your experience of going through cancer treatment?
    - *How easy/hard did you/ do you find it to get the care that you needed?*
    - *Can you think of any specific barriers you faced when seeking care and support for cancer?*

4. Can you think of any particularly helpful or positive experiences you have had with healthcare services? If so, what was it about the service/professional that made it a positive experience?

5. What do you think would improve health, and specifically cancer prevention, services? How could these have been made more accessible to you?
    - *What could be done to ensure that people experiencing homelessness are appropriately screened for cancer?*

6. What sorts of things do you currently do or would you like to do to improve your health and wellbeing?
    - *Prompt: Exercise, improve diet, reduce substance use, mindfulness, meaningful activity.*

7. As we work to develop a more tailored model of cancer prevention for people experiencing homelessness, do you think there are any particular factors that we need to consider?

8. Do you have anything else you would like to add to this discussion, or any questions you would like to ask me?

**Question schedule – Healthcare professionals.**

1. Could you start by briefing explaining your role, and the frequency and way in which you engage with people who are experiencing homeless?

2. From your experience, what are the typical health needs of people who are experiencing homeless?
    - *What effects do you see homelessness having on health?*
    - *What effects do you see homelessness having on healthcare access?*
    - *What specific health issues/concerns do homeless people tend to present with?*

3. Based on your experience, how would you characterise the current models/ approaches to cancer prevention for people experiencing homelessness in your area? Is there anything that is working particularly well or not working well?

4. Have you faced any particular barriers in engaging/caring for/treating homeless people in terms of cancer-related screening or treatment? (Individual, service or system related).
    - *How easy/hard do you find engaging/screening/diagnosing homeless people?*
    - *Do any examples come to mind?*

5. As we work to develop a tailored model of cancer prevention for people experiencing homelessness, do you think there are any particular factors that we need to consider? For example, in terms of their specific health or support needs?
    - *What strategies do you think would work in terms of improving engagement?*
    - *What could be done to ensure that people experiencing homelessness are appropriately screened for cancer?*
    - *Would you anticipate any obstacles in implementing a more co-ordinated model of care brings together health and social care services?*

6. Do you have anything else you would like to add to this discussion, or any questions you would like to ask me?

**Question schedule – Psycho-social care professionals.**

1. Could you start by explaining a little bit about your role, and the way in which you engage with people who are experiencing homeless?

2. From your experience, what are the typical health needs of people who are experiencing homeless?
    - *What effects do you see homelessness having on health?*
    - *What effects do you see homelessness having on healthcare access?*
    - *What specific health issues/concerns do people experiencing homelessness tend to present with?*

3. How would you describe your current knowledge of cancer?
    - *How confident would you be to offer cancer prevention advice/signposting to a client?*

4. What advice would you give a homeless person you were working if they were to present with a recognised symptom of cancer (or another health-related issue)? Where would you suggest they go to seek care/support?

5. What barriers/issues have you experienced, if any, when trying to support homeless people to access healthcare - and specifically cancer - related services?
    - *How easy do you find engaging homeless people with relevant services?*
    - *How would you characterise the accessibility of relevant services in this area?*
    - *What is your relationship with healthcare providers in the area?*
    - *Do any examples come to mind?*

6. Based on your experience, how would you characterise current models/ approaches to cancer prevention for people experiencing homelessness? Is there anything (services/approaches) that is working particularly well?

7. As we work to develop a tailored model of cancer prevention for people experiencing homelessness, do you think there are any particular factors that we need to consider? For example, in terms of their specific health or support needs?
    - *What strategies do you think would work in terms of improving engagement?*
    - *What could be done to ensure that people experiencing homelessness are appropriately screened for cancer?*

- *Would you anticipate any obstacles in implementing a more coordinated model of care brings together health and social care services?*

8. Do you have anything else you would like to add to this discussion, or any questions you would like to ask me?
